# Progress and Remaining Gaps in Estimating the Global Disease Burden of Influenza

**DOI:** 10.3201/eid2407.171270

**Published:** 2018-07

**Authors:** Joseph Bresee, Julia Fitzner, Harry Campbell, Cheryl Cohen, Vanessa Cozza, Jorge Jara, Anand Krishnan, Vernon Lee,, for the WHO Working Group on the Burden of Influenza Disease

**Affiliations:** Centers for Disease Control and Prevention, Atlanta, Georgia, USA (J. Bresee);; World Health Organization, Geneva, Switzerland (J. Fitzner, V. Cozza);; University of Edinburgh, Edinburgh, Scotland, UK (H. Campbell);; National Institute for Communicable Diseases, Johannesburg, South Africa (C. Cohen);; Universidad del Valle de Guatemala, Guatemala City, Guatemala (J. Jara);; All Indian Institute of Medical Sciences, New Delhi, India (A. Krishnan);; Ministry of Health, Singapore (V. Lee)

**Keywords:** influenza, epidemiology, surveillance, disease burden, influenza vaccines, pandemic influenza, World Health Organization, viruses, vaccines, respiratory infections

## Abstract

Influenza has long been a global public health priority because of the threat of another global pandemic. Although data are available for the annual burden of seasonal influenza in many developed countries, fewer disease burden data are available for low-income and tropical countries. In recent years, however, the surveillance systems created as part of national pandemic preparedness efforts have produced substantial data on the epidemiology and impact of influenza in countries where data were sparse. These data are leading to greater interest in seasonal influenza, including implementation of vaccination programs. However, a lack of quality data on severe influenza, nonrespiratory outcomes, and high-risk groups, as well as a need for better mathematical models and economic evaluations, are some of the major gaps that remain. These gaps are the focus of multilateral research and surveillance efforts that will strengthen global efforts in influenza control in the future.

Influenza has long been a global public health priority because of the ever-present threat of another global pandemic. In addition, many countries (especially in more affluent, temperate areas) prioritize influenza prevention and control programs because of the annual effects of seasonal influenza. The 3 influenza pandemics that occurred during the 20th century clearly illustrated the major impact from the global spread of a new influenza A virus (*1*) and spurred early vaccine development (*2*,*3*). The reemergence of avian influenza A(H5N1) in Asia in 2004 served as a reminder of this threat and brought about an acceleration of national and international efforts to prepare for the next pandemic (*4*). These efforts, including the expansion of influenza surveillance and laboratory capacity, contributed to a more effective response during the 2009 influenza A(H1N1) pandemic (*5*).

Although the threat of novel influenza A viruses and pandemics has mobilized national preparedness efforts, in many parts of the world the importance of seasonal epidemics of influenza has been relatively underappreciated. On the basis of findings from recent influenza respiratory mortality studies, including estimates from a study conducted by Iuliano et al (*6*), the World Health Organization (WHO) has indicated that 290,000–650,000 respiratory deaths from seasonal influenza epidemics occur annually (*7*). Most high-income countries, where substantial work to document the disease burden from annual influenza epidemics has been conducted, have longstanding and robust influenza vaccination programs (*8*); in these settings, the use of influenza antiviral drugs and antibiotics to treat influenza-associated lower respiratory tract infections is relatively routine. These strategies have likely resulted in a reduction in the burden of disease as well as improved clinical outcomes for patients with influenza. In tropical and low- and middle-income countries (LMICs), seasonal influenza has often been viewed as a disease of relevance primarily to industrialized countries. The historical paucity of data on influenza from these settings has likely contributed to this view. More data on influenza burden in these locations are needed to make compelling arguments to policy makers for investments in seasonal influenza control and prevention. These data are particularly important in the light of challenges related to the variable effectiveness of current vaccines (*9*) and the programmatic and economic difficulties in conducting annual influenza vaccination programs or in implementing appropriate use of antivirals for treatment in LMICs. The resulting relative underuse of vaccines in many LMICs represents important missed opportunities for disease prevention (*10*). Furthermore, the lack of antiviral drug treatment and influenza vaccination programs for reduction of seasonal influenza burden in these settings also jeopardizes the capacity for effective responses when the next pandemic emerges because national pandemic response plans rely, in part, on the timely and efficient use of medical countermeasures, such as antiviral drugs and vaccines for pandemic viruses (*11*,*12*).

## Progress, but Gaps Remain

The growth of influenza surveillance and research in the past 10 years has generated substantial new data on the epidemiology and risk from influenza around the world, notably in tropical countries and LMICs (*13*–*16*). These data have confirmed that influenza is a major cause of hospitalization and severe acute respiratory disease in all settings, whether rich or poor, tropical or temperate, urban or rural (*17*–*19*), and that the risk for severe influenza outcomes might be higher in LMICs than in high-income countries (*20*). In South Africa, for instance, rates of influenza-associated mortality among the elderly were 3–4 times higher than those among the elderly in the United States (*21*). One global estimate of childhood influenza deaths indicated that 99% of all influenza-associated deaths among children <5 years of age were in LMICs (*18*). That this finding is true for influenza, as it is for other infectious diseases, is not surprising, and similarly, is likely the result of differential access to medical care and preventive strategies, coupled with the prevalence of certain high-risk conditions and the underlying age structure of the populations. In some LMICs that have collected robust disease burden information in the past decade, vaccine programs have expanded (*22*–*24*). In addition, disease burden data from these settings were one driving factor for updating of the WHO influenza vaccine recommendations issued in 2012 by the WHO Strategic Advisory Group of Experts (*25*).

However, although data on influenza disease burden have expanded in recent years, considerable gaps persist. First, high-quality and up-to-date estimates of the extent of severe influenza at global and regional levels are needed to inform global policymakers and public health advocates as they set their priorities. Although new estimates of the global respiratory mortality rates attributable to influenza are available (*6*), additional models that take advantage of the expansion in influenza surveillance and laboratory confirmation, especially in tropical countries and LMICs, should yield more accurate country- and region-specific disease estimates. Second, at the country level, too many LMICs have yet to develop reliable national estimates of the full extent of influenza disease that would enable evidence-based decisions about local influenza prevention investments. Third, the ability to target vaccination campaigns to key populations within a country depends on having reliable data on the burden of disease and on the possible effect of vaccination among specific high-risk target groups. The value of risk group–specific estimates was evident during the 2009 pandemic, when data on the high risk for severe outcomes among pregnant women led to aggressive efforts to vaccinate and appropriately treat this group and convince obstetricians to recommend and offer vaccines (*26*,*27*); this effort provided data for the WHO Strategic Advisory Group of Experts’ 2012 recommendations (*25*). Conversely, the scarcity of adequate data on severe disease among pregnant women during seasonal epidemics was one reason cited by the Global Alliance for Vaccines and Immunizations for their decision against opening an investment window to fund low-income countries to vaccinate pregnant women as part of their most recent vaccine investment strategy (*28*). Data have long indicated that persons with specific underlying diseases are at high risk for severe influenza, but without a better understanding of the burden of the disease in these groups in countries considering vaccination policies, expecting policymakers to invest in programs to target them is unrealistic. Few data have been collected outside of high-income countries on other components of the health burden, especially the contribution of influenza infections to illness and death from underlying diseases made worse by influenza, such as cardiac or chronic pulmonary diseases (*29*), and on non–health-related effects of influenza, such as the economic burden and effect on productivity (*30*). Data on each of these components will advance decisions on the rational use of resources to prevent influenza, and the need for these data was highlighted in the recent revision of the WHO Influenza Research Agenda (*31*).

## Ongoing Work to Address the Gaps and Future Needs

Substantial work is under way to fill these gaps. WHO has created a robust program to collect data on global and national influenza burden and to better determine the burden among risk groups. Two manuals have been developed to guide member states’ efforts to measure influenza disease (*32*) and economic burden (*33*) from data collected through ongoing influenza surveillance. Both manuals are being used by countries, in part, because of the Pandemic Influenza Preparedness Implementation Plan that has facilitated country-level disease burden estimation in many countries around the world (*34*). These efforts have led to recent publications from LMICs, supported by WHO, that provide important influenza disease burden data (*34*). WHO has also sponsored reviews of influenza-associated disease burden among pregnant women and their infants (*35*,*36*). Additional multinational collaborations are under way that will continue to develop more credible global influenza mortality and hospitalization estimates based on recent work to develop national estimates, as well as information on influenza burden among key high-risk groups (Figure) (*6*). These efforts take advantage of the recent increase in local- or country-level studies that have extended data beyond temperate, high-income settings. WHO is also mapping existing knowledge from published literature to enable easier access to available data and identify key remaining gaps. Finally, WHO is developing a collection of economic tools to support the use of disease burden data to estimate the overall costs, the cost of vaccination programs, and the cost-effectiveness of vaccination.

**Figure:**
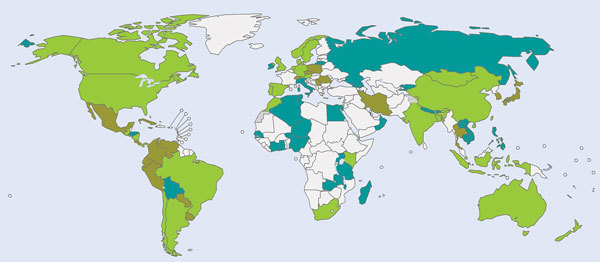
Countries with burden of disease estimates that have agreed to share data for the global estimate of influenza mortality and influenza-associated hospitalizations, as of April 2017: blue, morbidity estimates; brown, mortality estimates; green, morbidity and mortality estimates; white, data not available.

In addition, other global partners are working in this area. The US Centers for Disease Control and Prevention has established collaborations with >50 countries around the world to strengthen surveillance and laboratory testing capacities, including expanding global capacity for genetic sequencing, that have produced data on influenza epidemiology and disease burden. The Multinational Influenza Seasonal Mortality Study (*37*), coordinated by the Fogarty Center at the US National Institutes of Health, has been working with countries to estimate influenza mortality from diverse settings since 2001. Institute Pasteur and Agence de Médecine Préventive have worked in low-income countries in Africa to build surveillance capacity. More recently, the Global Health Security Agenda has increased resources to many developing countries to strengthen surveillance and response capabilities that will also lead to new data on the relative burden of influenza compared with other infectious diseases. The European Center for Disease Control has developed free software that supports countries in the region to estimate the burden of influenza and other infectious diseases (*38*). These efforts have led to a substantial increase in country-specific estimates of burden in the past 5 years and promise to lead to many more in the next 1–2 years.

Much has been accomplished, and in the next few years a more complete picture of the burden of influenza will be available. However, more work will still be needed if we are to measure the full burden of influenza and, more important, the preventable burden. This knowledge will enable decision-makers to weigh the value of vaccination and encourage the use of antiviral drugs against myriad other health needs in their countries, as well as providing additional impetus for the development of newer, more effective treatments and vaccines. Vaccine probe studies have been proposed as a method to measure the preventable fraction of disease burden, focusing the studies on outcomes of greatest public health interest (e.g., pneumonia and death rates). Whether vaccine probe studies could be designed sufficiently to account for the variable and relatively modest vaccine efficacy and variability in annual disease burden that is characteristic of influenza is uncertain but should be further discussed (*39*). Finally, the year-to-year variability in disease burden requires that data be collected over multiple years and that new methodologic approaches be developed and validated to measure burden in settings with year-round circulation of influenza (*40*)

## Conclusions

Influenza has long been a compelling example of a global pandemic threat, but the annual disease burden has been relatively underappreciated, leading to missed opportunities for disease reduction and prevention. Convincing evidence of seasonal burden of disease in more settings and for a wider array of influenza outcomes will be the foundation of arguments for strengthening programs to control annual influenza and to reduce the threat of future pandemics (*41*,*42*).
